# Lumbar Pseudogout Mimicking Pyogenic Spondylitis in an Older Patient: A Diagnostic Pitfall in Emergency Care

**DOI:** 10.7759/cureus.100965

**Published:** 2026-01-06

**Authors:** Masaatsu Kuwahara, Hideaki Imanaka

**Affiliations:** 1 Emergency Medicine, Takarazuka City Hospital, Takarazuka, JPN; 2 Critical Care Medicine, Takarazuka City Hospital, Takarazuka, JPN

**Keywords:** calcium pyrophosphate deposition disease, inflammatory markers, joint fluid analysis, low back pain, lumbar pseudogout, pyogenic spondylitis

## Abstract

Lumbar pseudogout, or calcium pyrophosphate dihydrate deposition disease (CPPD), is an uncommon manifestation of a crystal-induced arthropathy that typically affects peripheral joints. Spinal involvement, particularly in the lumbar region, is rare and may closely mimic infectious conditions such as pyogenic spondylitis.

An 85-year-old man was hospitalized for pneumococcal pneumonia and subsequently developed severe low back pain on day 7 of admission. Although his pneumonia improved with antimicrobial therapy, inflammatory markers remained elevated, raising suspicion of pyogenic spondylitis. Computed tomography and magnetic resonance imaging of the lumbar spine demonstrated compression fractures and inflammatory changes at the L2/3 intervertebral disc but were insufficient to establish a definitive diagnosis. A diagnostic intervertebral disc biopsy was therefore performed, and polarized light microscopic examination revealed calcium pyrophosphate dihydrate crystals, confirming the diagnosis of lumbar pseudogout. Nonsteroidal anti-inflammatory drugs were discontinued because of renal dysfunction, and the patient’s symptoms improved rapidly after corticosteroid therapy.

This case highlights the diagnostic challenge of lumbar pseudogout, which can closely resemble infectious or neoplastic spinal disease. Although imaging plays an important role in excluding alternative diagnoses, definitive diagnosis of CPPD relies on crystal identification, most commonly through synovial fluid analysis or, when not feasible, tissue biopsy. Lumbar pseudogout should be considered in older adults presenting with acute inflammatory low back pain to facilitate timely diagnosis and appropriate management.

## Introduction

Lumbar pseudogout, or calcium pyrophosphate dihydrate deposition disease (CPPD), is a crystal-induced arthropathy caused by the deposition of calcium pyrophosphate crystals in articular cartilage, fibrocartilage, ligaments, and other periarticular tissues. Since its first description in 1962, CPPD has been recognized as a common condition, particularly in older adults, and it most commonly affects peripheral joints such as the knees and wrists. In contrast, spinal involvement is uncommon; most reported cases involve the cervical spine, particularly in the form of crowned dens syndrome. Lumbar involvement is considerably rarer and remains underrecognized in clinical practice [1,2].

Lumbar CPPD predominantly affects older adults and presents with a wide range of symptoms, including acute low back pain, sciatica, fever, and neurologic deficits. Unlike gout, which is caused by monosodium urate crystal deposition and more commonly affects peripheral joints, CPPD-related spinal disease often lacks characteristic clinical features. It may closely mimic infectious or neoplastic conditions. Because clinical manifestations and imaging findings frequently resemble those of pyogenic spondylitis, metastatic spinal tumors, lumbar disc herniation, or spinal canal stenosis, establishing an accurate diagnosis is often challenging [3,4].

Although computed tomography (CT) and magnetic resonance imaging (MRI) may demonstrate calcifications or mass-like lesions within the intervertebral discs, facet joints, or ligamentum flavum, these findings are nonspecific, and differentiation from infectious or malignant etiologies remains difficult. Delayed or incorrect diagnosis may result in unnecessary antimicrobial therapy or invasive interventions; therefore, recognition of lumbar CPPD as a potential differential diagnosis is clinically significant, particularly in older patients presenting with acute inflammatory back pain.

This report describes a case of lumbar pseudogout in an 85-year-old man who developed severe low back pain during treatment for pneumococcal pneumonia.

## Case presentation

Patient background and chief complaint

An 85-year-old man with a medical history of hypertension, left breast cancer (luminal A subtype without metastasis), currently treated with oral tamoxifen, and lumbar compression fractures was admitted to our hospital for treatment of pneumococcal pneumonia (A-DROP score, 2). Although his pneumonia improved with antimicrobial therapy, his low back pain gradually worsened during hospitalization.

Current medical history

On admission, the patient tested positive for pneumococcal antigen in urine and was diagnosed with pneumococcal pneumonia. *Streptococcus pneumoniae* was later identified on sputum culture. Treatment consisted of intravenous ceftriaxone (2 g/d) for seven days and oral azithromycin (250 mg/d) for three days. His respiratory status improved; however, on day 7 of hospitalization, his low back pain progressively worsened.

Differential diagnoses included pyogenic spondylitis, a new lumbar compression fracture, and metastatic breast cancer. Because blood tests showed elevated inflammatory markers, pyogenic spondylitis was considered the most likely diagnosis, and further evaluation was pursued.

Physical examination

Physical examination revealed localized tenderness over the midline lumbar region. Neurological examination of the lower extremities, including assessment of muscle strength, sensory function, deep tendon reflexes, and straight leg raising, revealed no focal neurologic deficits.

Laboratory and imaging findings

**Figure 1:**
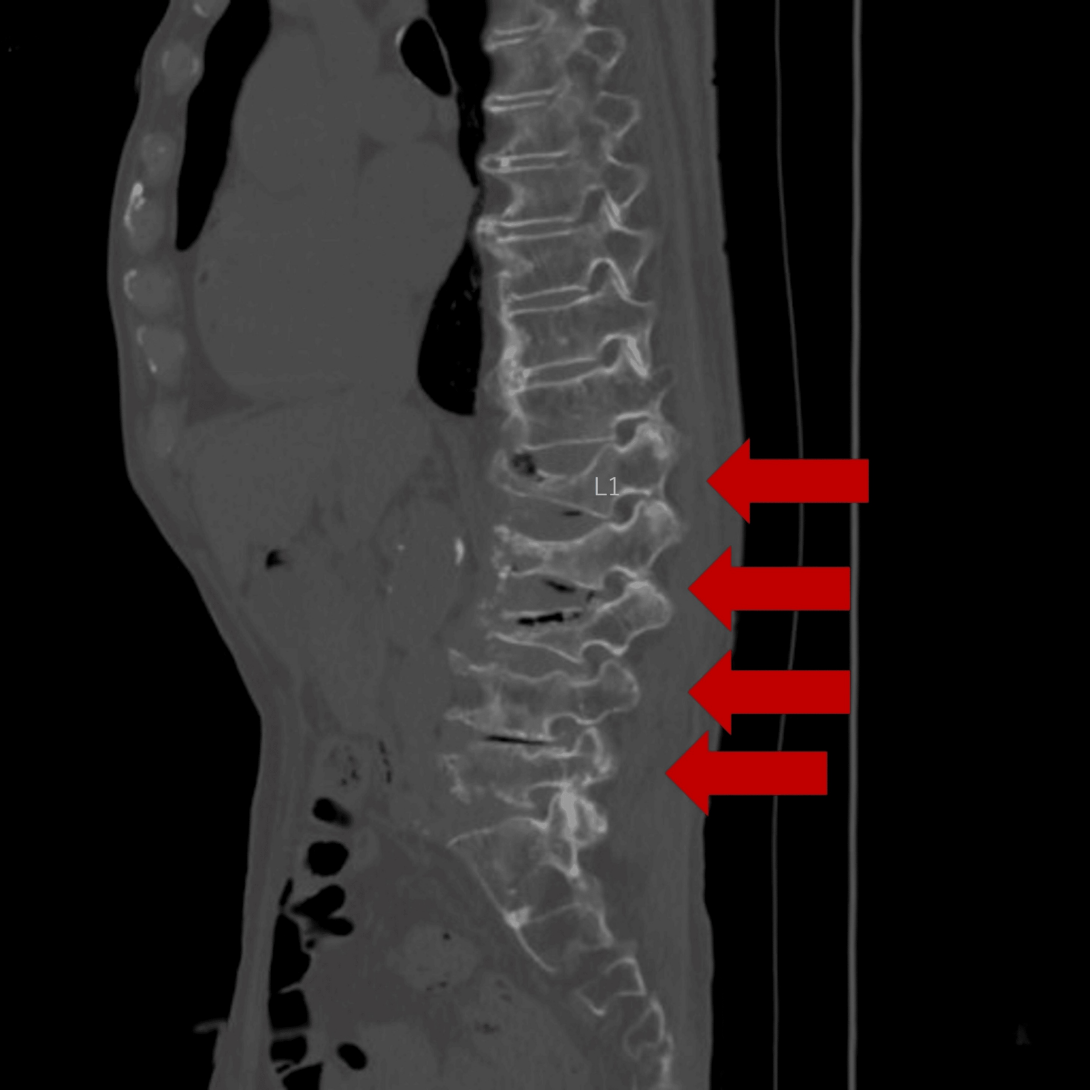
Lumbar spine CT Compression fractures were identified from Th12 to L5 (arrows). CT: computed tomography

**Figure 2:**
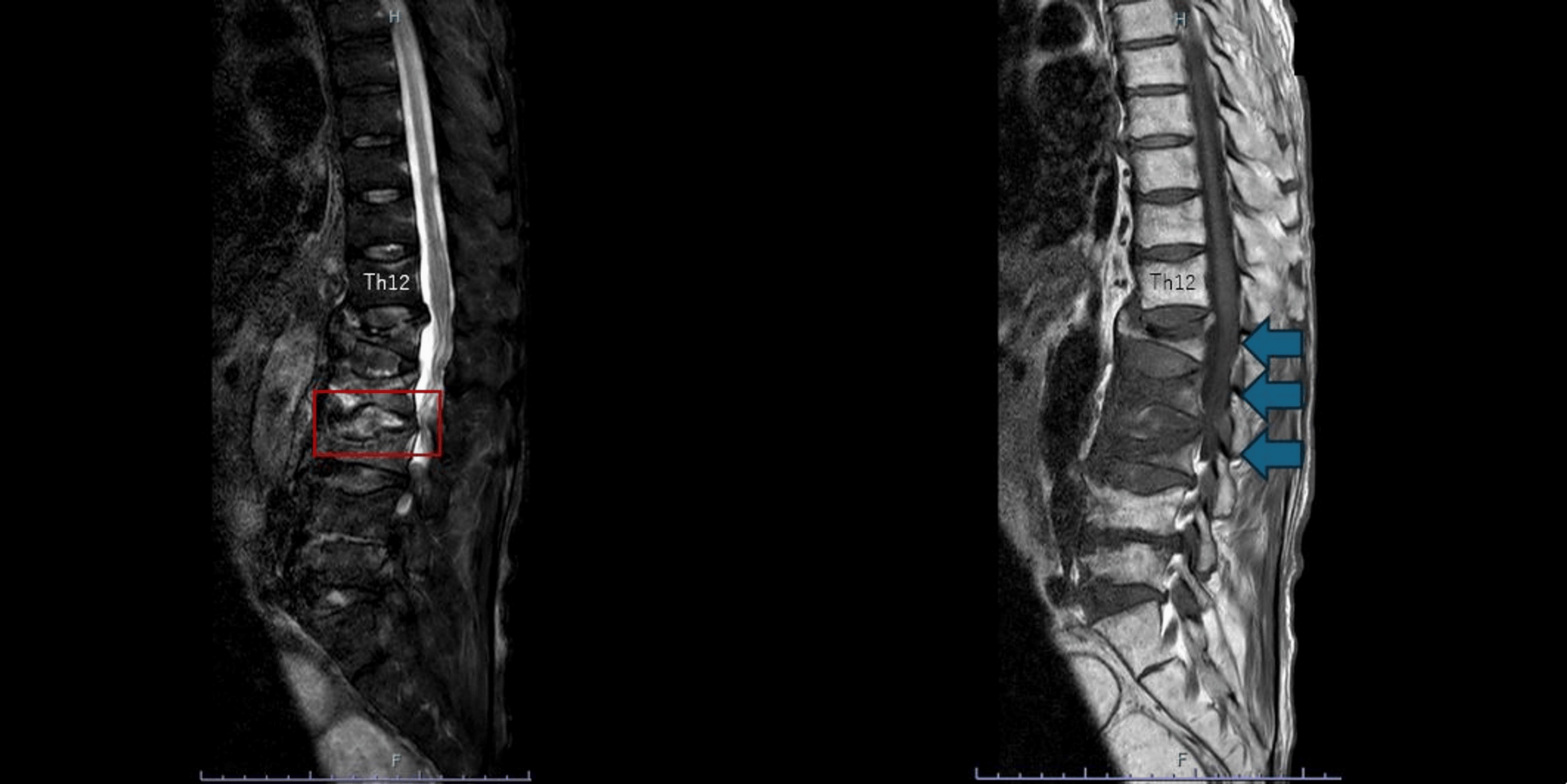
Lumbar spine MRI showing T2-weighted fat-suppressed images (left) and T1-weighted images (right) On T2-weighted fat-suppressed images, the L2/3 intervertebral disc demonstrates high signal intensity. On T1-weighted images, decreased signal intensity of the bone marrow from L1 to L3 is observed, consistent with acute compression fractures. MRI: magnetic resonance imaging

Laboratory testing demonstrated a white blood cell count of 12,610/µL (reference range, 3360-8800/µL) with a neutrophil fraction of 83.5%, indicating a left shift. The C-reactive protein level was 14.66 mg/dL. Blood cultures obtained at the time of exacerbation of low back pain were negative. A CT of the lumbar spine (Figure 1) and an MRI of the lumbar spine (Figure 2) were performed as part of the diagnostic evaluation.

MRI demonstrated findings suggestive of acute compression fractures as well as inflammatory changes at the L2/3 intervertebral disc. Because pyogenic spondylitis could not be excluded based on these imaging findings, a diagnostic intervertebral disc biopsy was performed.

Treatment and clinical course

Given the elevated inflammatory markers and imaging findings, an open intervertebral disc biopsy was performed. Histopathological examination and microbiological cultures of the aspirated material showed no bacterial growth; however, calcium pyrophosphate dihydrate crystals were identified under polarized light microscopy following routine staining. These findings established the diagnosis of lumbar pseudogout.

Because Japan is considered an intermediate tuberculosis burden country, tuberculosis was included in the differential diagnosis; however, tuberculosis-specific interferon-γ release assays were negative, effectively excluding tuberculous arthritis.

Initial treatment consisted of oral nonsteroidal anti-inflammatory drugs (indomethacin, 200 mg/d for seven days). Although the patient’s pain showed a tendency toward improvement, continuation of NSAIDs was deemed unsafe because he subsequently developed renal dysfunction, with blood urea nitrogen increasing from 28.2 mg/dL (reference range, 8-20 mg/dL) to 45.2 mg/dL and serum creatinine rising from 1.82 mg/dL (reference range, 0.65-1.07 mg/dL) to 2.73 mg/dL. Therapy was therefore switched to intramuscular triamcinolone acetonide (40 mg, single dose), resulting in rapid improvement of symptoms.

## Discussion

Diagnostic challenges

In this case, diagnostic complexity arose from the overlapping clinical presentations of lumbar pseudogout and pyogenic spondylitis. Both conditions may present with severe back pain and elevated inflammatory markers, making differentiation difficult. The patient’s recent history of pneumococcal pneumonia further complicated the clinical picture and initially supported suspicion of infectious spondylitis.

Role of imaging

CT and MRI each have distinct advantages and limitations in evaluating spinal disorders. CT is widely available and effective for assessing osseous structures, vertebral deformities, and calcifications. In contrast, MRI provides superior soft-tissue contrast and is essential for evaluating bone marrow edema and inflammatory changes.

In the present case, these modalities were complementary: CT contributed to the assessment of vertebral morphology and the exclusion of gross structural abnormalities, whereas MRI was required to evaluate disc involvement and to assess for acute fracture and metastatic disease. Nevertheless, even with combined imaging, differentiation between pyogenic spondylitis and crystal-induced inflammatory disease remained difficult, necessitating histopathological confirmation.

Importance of crystal identification

Previous literature indicates that although imaging modalities may suggest CPPD, definitive diagnosis generally relies on identification of calcium pyrophosphate dihydrate crystals under polarized light microscopy, most commonly in synovial fluid, which remains the diagnostic gold standard when clinical or imaging findings are inconclusive [5-8].

In the present case, synovial fluid analysis was not feasible because the lesion was confined to the intervertebral disc. Instead, a diagnostic intervertebral disc biopsy was performed, and polarized light microscopic examination of the biopsy specimen demonstrated calcium pyrophosphate dihydrate crystals, thereby establishing the diagnosis of lumbar pseudogout.

Treatment and prognosis

The primary treatments for pseudogout include nonsteroidal anti-inflammatory drugs and colchicine. In the present case, continuation of NSAIDs was not feasible because of renal dysfunction; however, the patient’s symptoms improved rapidly after initiation of systemic corticosteroid therapy. Although spontaneous improvement cannot be excluded in a single case, corticosteroids appeared to be clinically effective in this patient. Previous reports have described favorable responses to corticosteroid therapy in patients with severe pseudogout or in those for whom NSAIDs are contraindicated [9,10].

## Conclusions

This case highlights the importance of considering lumbar pseudogout in older adults presenting with acute low back pain and elevated inflammatory markers, particularly when pyogenic spondylitis is suspected. Although imaging studies help exclude alternative diagnoses, the definitive diagnosis of CPPD relies on identification of calcium pyrophosphate crystals, most commonly via synovial fluid analysis. In the present case, the diagnosis was established by crystal identification in an intervertebral disc biopsy specimen. Awareness of this diagnostic principle may facilitate timely diagnosis and appropriate management of lumbar pseudogout.
